# Does Repeated Ticking Maintain Tic Behavior? An Experimental Study of Eye Blinking in Healthy Individuals

**DOI:** 10.3233/BEN-120302

**Published:** 2013

**Authors:** Daniel J. V. Beetsma, Marcel A. van den Hout, Iris M. Engelhard, Marleen M. Rijkeboer, Danielle C. Cath

**Affiliations:** ^1^Altrecht Academic Anxiety CenterUtrechtThe Netherlands; ^2^Department of Clinical and Health PsychologyUtrecht UniversityUtrechtThe Netherlands

**Keywords:** Tourette syndrome, tics, eye blink, experimental model, human model

## Abstract

Tics in Tourette Syndrome (TS) are often preceded by 'premonitory urges': annoying feelings or bodily sensations. We hypothesized that, by reducing annoyance of premonitory urges, tic behaviour may be reinforced. In a 2X2 experimental design in healthy participants, we studied the effects of premonitory urges (operationalized as air puffs on the eye) and tic behaviour (deliberate eye blinking after a puff or a sound) on changes in subjective evaluation of air puffs, and EMG responses on the m. orbicularis oculi. The experimental group with air puffs+ blinking experienced a decrease in subjective annoyance of the air puff, but habituation of the EMG response was blocked and length of EMG response increased. In the control groups (air puffs without instruction to blink, no air puffs), these effects were absent. When extrapolating to the situation in TS patients, these findings suggest that performance of tics is reinforced by reducing the subjective annoyance of premonitory urges, while simultaneously preventing habituation or even inducing sensitisation of the physiological motor response.

## 1. Introduction

Gilles de la Tourette's Syndrome (TS) is a tic disorder with a lifetime prevalence of 0.8% [1]. Tics involve rapid, sudden, repetitive, and abnormal movements or vocalizations. Motor tics often involve musculature in the face and the neck, such as eye blinking or head shaking, and vocal tics vary from throat clearing and coughing to more complex variants like coprolalia – involuntary swearing or uttering obscene words [2]. TS is partly genetically determined [3]. At present, TS is understood as a neuropsychiatric disorder, of which symptoms are best alleviated by either behaviour therapy or pharmacotherapy.

Tics in TS usually do not occur out of the blue. Over 90% of adult TS patients report that executing a tic provides relief from sensory sensations commonly described as *premonitory urges*. Patients describe these urges as annoying physical sensations, or feelings of incompleteness or 'energy', which precede the tic [4, 5]. As a consequence, it is reasonable to assume that tic behaviour is rewarded by negative reinforcement: the tic results in the diminishing or disappearance of unpleasant sensations. However, this strategy might become less effective or even counterproductive after repeated ticking. With regard to the latter option, already in 1902, Meige and Feindel wrote: *"the more a tic is repeated, the more inveterate it becomes and the greater the likelihood of its becoming generalized"* [6]. The question arises whether the hypothetical initial rewarding effect of repetitive ticking (relief from and decrease of premonitory urges) diminishes with time, and might paradoxically lead to an increase in the inten-

ISSN 0953-4180/13/$27.50 © 2013 – IOS Press and the authors. All rights reserved sity and frequency of tics to maintain the initial relief from the urges. This would be analogous to the long-term reinforcing effects of repetitive behaviours in other conditions characterised by both impulsive and compulsive actions, such as obsessive-compulsive disorder (OCD) and addictive behaviour. The reinforcing effect of repetitive compulsions has been thoroughly studied in experimental designs of OC behaviour, a TS-related condition [7,8]. OCD is characterised by persistent obsessions (recurrent intrusive thoughts) which are followed by compulsions (repetitive behaviours performed according to a certain set of rules, to neutralize the anxiety/tension-provoking obsessions [9]. Various experiments in normal subjects showed that compulsive checking, e.g.: repetitive switching on and off of light switches or gas knobs results in *less* memory certainty of whether the object checked (gas or light) has been turned off [7,10], thus fuelling even more checking behaviour. This research has several implications: first, it provides an explanation of one of the factors that might maintain OC behaviour, and second it provides clinicians with a rationale to encourage their patients to limit their compulsions and tolerate the anxiety-provoking obsessions that accompany them, instead of engaging in a struggle to neutralize the latter. Similar factors as involved in the maintenance of OC behaviours might apply to the maintenance or aggravation of TS symptoms; tic behaviour in itself might fuel subsequent tic behaviour.

The hypothesised effects of tic behaviour on premonitory urges and on the intensity of future tics, anticipated by Meige and Feindel, (see above) should preferably be tested under well controlled laboratory conditions in which premonitory urges and ticking are experimentally induced. Three types of experimental subjects may be envisaged: animals, healthy participants or TS patients. An obvious problem using animals is that subjective experiences like premonitory urges cannot be assessed. Moreover, generalisability of findings across species and clinical validity may be problematic. Using afflicted (TS) patients may at first sight seem to be clinically most relevant, but there is the fundamental methodological problem that TS patients display, by diagnostic definition, habitual tic behaviour. It would be unclear if the effects of experimentally induced tic behaviour would result from the experimental manipulation itself or from an interaction with effects of a long history of ticking or other co-morbid repetitive behaviour of TS patients. To test the effects of premonitory urges and of subsequent ticking, in and by themselves, we decided to study the effects of an experimental model of *de novo* ticking and premonitory urges in healthy participants, with no history of TS.

Given that the most prevalent premonitory urges and tics relate to the eye blink [11], we induced an urge to blink by inducing sensations of dryness and itchiness to the eye, using air puffs as a proxy of the premonitory unpleasant itching sensations and urges to blink. The amplitude and duration of the induced blink reactions, considered as an acceptable operationalisation of tics, were assessed using EMG.

The hypotheses were tested whether, compared to relevant control conditions (see below), a period of deliberate eye blinking in reaction to air puffs to the eye will (a) subjectively decrease the unpleasant sensations induced by the air puffs, and in the meanwhile (b) increase responses of the m. orbicularis oculi to future air puffs.

## 2. Method

### 2.1. Participants

Ninety healthy undergraduate students of Utrecht University (54 females; mean age 22.4 years, SD = 2.2) participated in exchange for course credit or a small remuneration. Twenty three participants were excluded as they wore or had worn contact lenses for three months or more, or had a personal or family history of tics.

### 2.2. Study design

As a proxy for "premonitory urges", half of the participants were given air puffs to their left eye to create an urge to blink. As the air puffs in the Puff conditions were necessarily accompanied by a soft click (produced by the air-valve, see below), participants in the No air puff-condition were also presented with this sound. As a proxy for 'tics', half of the participants of both groups were asked to deliberately eye blink when perceiving the puff or sound, by firmly squeezing both eyes. The other half of the participants did not receive any response instructions. So, participants were randomized to one of four groups: group 1 (Puff + Blink) received an air puff and instruction to blink; group 2 (Puff + No blink) received an air puff without instruction to blink; group 3 (No puff + Blink) received no air puff, but was instructed to blink when the clicking sound was presented; group 4 (No puff + No blink) received no air puff and no instruction to blink.

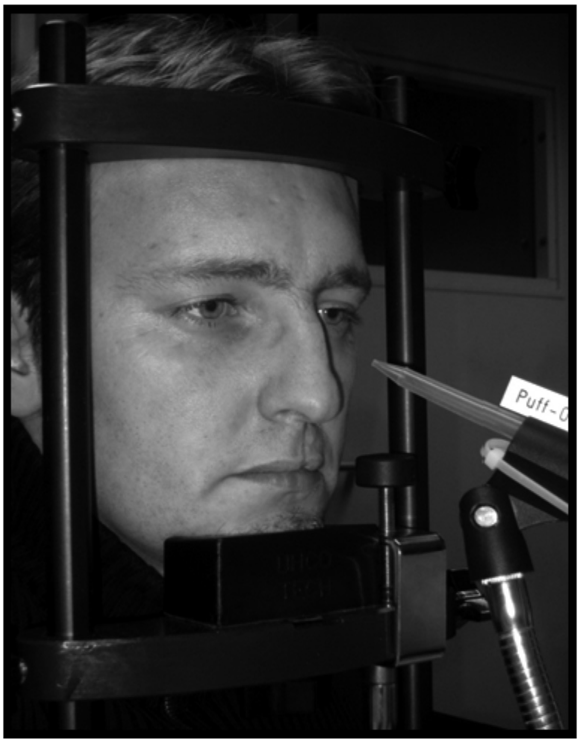

Fig. 1. Test situation: Participants placed their heads in a black metal headrest.

### *2.3. Materials and assessments*

#### *2.3.1. Air puff-device*

Air puffs were delivered by a modified Kooltronic V64 cooker hood air turbine. Using a plastic tube, the exhaust pipe was connected to a valve that could open and close, which transformed the constant air pressure into discrete air puffs. This valve, a Teflon high purity Valcor Scientific type SV51C56T34-8 by Inacom Instruments, was controlled by E-Prime 2.0®. From the valve, a second tube with a diameter of 80 mm transported the air puffs to a conical hard-plastic tip with a 1.5-mm diameter opening directed at the participant's left eye. Distance between the end of the tip and the eye was approximately 2 cm (see Fig. 1).

The strength of the puff was piloted and set at a level where EMG responses seemed reliable but not too high to overrule experimental effects.

#### *2.3.2. Blink reflex (EMG)*

Before and after the experiment, the strength of the eye blink response to puffs was recorded at the m. orbico orbicularis (m.OO) following published guidelines [12]. After skin preparation with water, two 8-mm electrodes were placed on the m.OO using Lectron II conductivity gel. One 12-mm grounding electrode was applied to the forehead. Signals were recorded by a Coulbourn isolated bio amplifier type V75-04 with bandpass filter (amplifier coupling: 1.0 Hz; gain: 10 k, high pass: 13 Hz; low pass: 150 Hz). Signals were analysed with Startle Analyser v10.20, which normalized the signals and calculated blink amplitudes. These were defined as the difference between the highest voltage reached in the 30–120 ms time span (corresponding with a R2 component of the blink reflex) and baseline voltage (i.e., the average voltage in the −40 ms/+ 10 ms time span (with 0 ms being stimulus onset). Pre-to-post change *ratios* were calculated for each participant, using the following formula:

$$\text{Blink reflex ratio} = \frac{\text{Average of } post \text{ manipulation blink amplitudes}}{\text{Average of } pre \text{ manipulation blink amplitudes}}$$

In addition to the blink reflex ratio as an outcome measure, a second EMG outcome measure was the *length* of the R2 component, defined as time elapsed between R2 onset and R2 offset.

#### *2.3.3. Subjective evaluations*

Before and directly after the experiment, participants evaluated the subjective nature of the puffs by completing three items using a 0–100 visual analogue scale (VAS): 1) how *annoying* were the puffs (0 = 'not annoying', 100 = 'very annoying'), how *strong* they perceived the puffs to be (0 = 'unnoticeable', 100 = 'very strong'), and how strong they experienced the *urge to blink* (0 = 'no urge', 100 = 'very strong urge').

#### *2.3.4. Headrest*

To prevent head movements from interfering with the EMG measurements, and to secure a constant distance to the puff valve, the participant's head was fixated in a headrest. Approximately 40 cm in front of the participants, a distraction picture with neutral content was placed, to divide the subjects from anticipation to possible future puffs.

### *2.4. Procedure*

#### *2.4.1. Pre-test*

With the electrodes in place, participants placed their chin on the headrest and were asked to focus on the distraction picture. The air puff device produced ten air puffs of 200 ms. Interstimulus interval (ISI) varied randomly between 3000 and 5500 ms. Electrical activity of the m. orbicularis oculi was recorded and, after the tenth puff, participants completed the first set of VAS scores.

Table 1
Means and standard deviations of change scores on the three VAS items

| Mean change scores on VAS items (SD) | | | | | |
|---|---|---|---|---|---|
| | | Puff | | No puff | |
| Blink | Intensity | −5.33 (4.57) | Intensity | 0.56 (4.34) |
| | Annoyance | −15.03 (3.82) | Annoyance | −1.38 (3.90) |
| | Urge | −33.18 (5.24) | Urge | −24.42 (6.13) |
| No blink | Intensity | −5.28 (5.37) | Intensity | −2.94 (4.49) |
| | Annoyance | 3.03 (5.99) | Annoyance | 0.85 (3.97) |
| | Urge | −13.54 (4.90) | Urge | −9.24 (4.51) |

*2.4.2. Experimental conditions*

In the Puff conditions, participants received 30 air puffs with a 200-ms pulse duration, again with ISI varying randomly between 3000 and 5500 ms. The puffs coincided with a soft but salient sound produced by the opening of the valve. In the No puff conditions, the valve was opened, which produced the sound, but puffs were not administered. In the Blink conditions, participants were instructed to firmly but briefly (slightly less than 1 s) squeeze both eyes upon perceiving the puff or tone. In the No blink conditions, participants received no instructions about how to respond to the puff or sound.

*2.4.3. Post-test*

The post-test was identical to the pre-test, with the exception that participants in the Blink conditions were told that squeezing the eyes in response to sounds or puffs was no longer required.

## 3. Results

*3.1. Subjective evaluations*

Pre-test to post-test differences in VAS scores were calculated and subjected to a 2 X 2 between groups ANOVA with Puff (puff vs. no puff) and Blink (blink vs. no blink) as independent factors.

For the pre-post changes in puff *annoyance*, there was no main effect of Puff ($F$ (1,62) = 1.66, $p$ = n.s., $\eta_p^2 = 0.03$). However, there was a main effect of Blink, $F$ (1,62) = 5.18, $p = 0.01$, one tailed, $\eta_p^2 = 0.08$, indicating that in the Blink conditions, annoyance ratings decreased more than in the No blink conditions. Furthermore, the annoyance of the puffs tended to decrease only if the blinks were performed in direct response to puffs (Puff + Blink), as reflected in a borderline significant Puff x Blink interaction, $F$ (1,62) = 3.15, $p = 0.08$, $\eta_p^2 = 0.05$. Importantly, post-hoc $t$-tests showed that in the Puff + Blink condition, the average change in annoyance differed significantly from zero, $t$ (14) = 3.94, $p < 0.001$, while in the other three conditions it did not, all $t$s < 0.51, all $p$s > 0.62. Thus, only in the experimental Puff + Blink condition annoyance was reduced. See Fig. 2 and Table 1 for means and standard deviations of change scores.

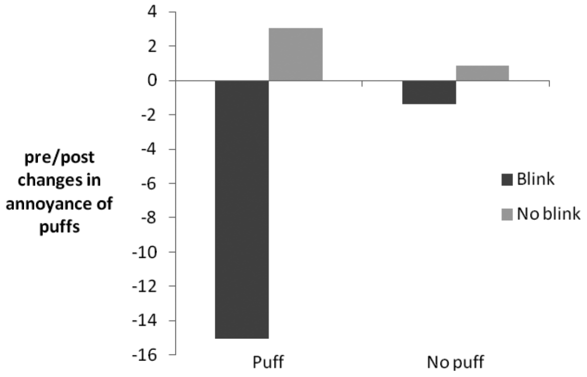

Fig. 2. Pretest to posttest changes in annoyance of puffs after tics/air puffs.

For the subjective change scores of puff *intensity*, there were no main effects for Puff, $F$ (1,62) = n.s., $p$ = n.s., $\eta_p^2 = 0.01$, or for Blink, $F$ (1,62) = 0.13, $p$ = n.s., $\eta_p^2 = 0.03$, nor was there any significant Puff x Blink interaction, $F$ (1,62) = 0.14, $p$ = n.s., $\eta_p^2 = 0.00$.

For changes in the perceived *urge* to blink, there was no main effect for Puff, $F$ (1,62) = 1.54, $p$ = n.s., $\eta_p^2 = 0.02$, but there was a significant main effect for Blink, $F$ (1,62) = 11.0, $p = 0.002$, $\eta_p^2 = 0.15$, indicating that in the Blink conditions the urge to blink decreased more than in the No blink conditions. There was no difference in effect between the two Blink conditions, and therefore as expected, the Puff x Blink interaction was not significant, $F$ (1,62) = 0.18, $p$ = .n.s., $\eta_p^2 = 0.00$.

*3.2. EMG*

Sixteen of the 66 participants failed to show the defined EMG responses in at least 3 of the 10 pre-test trials. EMG analyses were repeated after they were excluded.

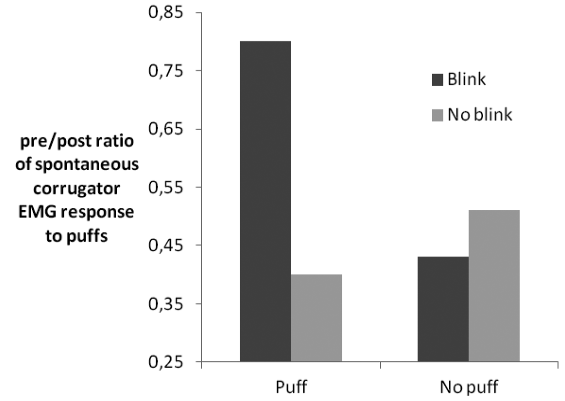

Fig. 3. Pretest to posttest ratio of corrugator EMG response to puffs (Blink reflex ratio).

### 3.2.1. *Amplitude of blink reflex* (*EMG*)

Figure 3 shows data on the ratios of the amplitude of the blink reflex.

There was no main effect for Puff, $F$ (1,49) = 0.01, $p$ = n.s., $\eta_p^2 = 0.00$, or Blink, $F$ (1,49) = 0.93, $p$ = n.s., $\eta_p^2 = 0.02$, on the amplitude of the blink responses between the groups. However, Fig. 3 shows that the highest Blink reflex ratios were observed in the Puff + Blink condition. This was reflected in a significant Puff x Blink interaction, $F$ (1,49) = 5.24, $p = 0.026$, $\eta_p^2 = 0.10$. Post-hoc paired t-tests showed that for the No puff conditions, there was no difference between Blink and No blink groups, $t$ (24) = 0.96 (ns). For the Puff conditions, those who blinked in response to puffs had a significantly higher Blink reflex ratio compared to those who were not instructed to blink, $t$ (25) = 2.26, $p = 0.03$. Crucially, if the Blink reflex ratio would not have changed from pre-test to post-test, the ratio would have remained 1. We tested whether each of the four groups displayed a ratio that differed from 1. This was the case for all three control groups (No Puff + Blink, Puff + No blink, No puff + No blink) but not for the Puff + Blink condition. Thus, all three control groups displayed a significant and substantial habituation (all $t$s > 5.0, all $p$s < 0.001). No such effect occurred in the experimental group (Puff + Blink): $t$ (13) = 1.06, n.s., (see Fig. 3). Apparently, the Puff + Blink combination blocked habituation of the EMG amplitude.

### 3.2.2. *Length of the R2 component* (*EMG*)

There was no main effect of Puff, $F$(1,48) = 2.39, $p$ = n.s., $\eta_p^2 = 0.05$, or Blink ($F$(1,48) = 1.29, $p$ = n.s., $\eta_p^2 = 0.03$) on length of the EMG R2 component between the groups. However, relative to the other conditions, in the Puff + Blink condition the length

Table 2
Means and standard deviations of changes in length of the R2 component

| | Mean changes in length of R2 component (SD) | |
|---|---|---|
| | Puff | No puff |
| Blink | 13.10 (6.27) | −2.97 (3.36) |
| No blink | −1.08 (4.75) | 0.71 (2.56) |

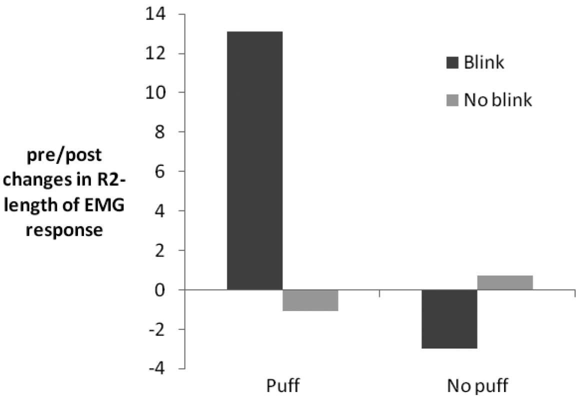

Fig. 4. Pretest to posttest changes in R2 length of corrugator EMG response to puffs.

of the R2 component of the blink reflex tended to be increased, as reflected by a borderline significant Puff x Blink interaction, $F$ (1,48) = 3.73, $p = 0.06$, $\eta_p^2$ = 0.07.

Post-hoc paired $t$-tests showed that for the conditions with puffs, the R2 component tended to be longer in the Blink group compared to the 'no blink' group: $t(25)$ = 1.78, $p = 0.08$. For No puff, this difference was not significant, $t(23) = 0.86$, n.s. Thus, the *combination* of Puff and Blink appears to provoke a prolongation of the duration of the EMG response.

## 4. Discussion

In this experimental study we tested in healthy participants whether intense and deliberate eye blinking in response to air puffs to the eye reduces subjective annoyance of the air flow but increases the intensity of the EMG response. Participants were randomized to four conditions: Blink (Y/N) vs. Puff (Y/N). Before and after the interventions, adversity of the puffs/urges was assessed using self-report (VAS's), and intensity of blinks was assessed by measuring the amplitude and duration of the EMG response at the m. orbico orbicularis. Only in the condition in which participants were instructed to blink in response to an air puff, they showed a significant drop in subjective annoyance of the puff. In addition, only in this crucial Puff + Blink condition, no habituation of the amplitude of the blink reflex occurred, while its duration even increased. The effects were rather specific. First, after instructed blinking, a decrease in puff annoyance was reported, but not in perceived intensity or the perceived urge to blink. Second, the effects were not due to merely the administration of puffs, because they did not occur in the Puff + No blink condition. Third, the effects were not due to blinking as such, since no effects occurred in the No puff + Blink condition. Apparently it was the *combination* of perceiving puffs and responding with blinks that fuelled the effects on EMG and self-reports.

The study was aimed to serve as a laboratory *model* of TS, and needs independent replication. Given the room that modelling provides for controlled study of cause-effect relationships and for quantification, formal/mathematical modelling or laboratory modelling (in animals or humans) provide powerful scientific tools. Still, the relevance of a model crucially depends on the to-be-modelled phenomenon and the isomorphic quality of the model: are the operationalisations of premonitory sensations and the subsequent blink response used in this experiment clinically valid to represent a tic model?

First, most tics encompass eye blink tics [11]. In this experiment eye blinks were induced by administration of air-puffs to the (left) eye, which reliably induced blinks. Second, like premonitory urges, the puff-induced sensations were unpleasant (annoying). The occurrence and strength of the blink was established using classical EMG assessment. Given these similarities between the created laboratory model and the clinical picture of TS, the model has the properties to inform clinical science about mechanisms that play a role in TS. A further confirmation of the validity of the present model is provided by our observations of the R2 component of the blink reflex which were in line with an early electrophysiological study in TS patients conducted by Smith and Lees [13]. In this study, TS patients showed, compared to healthy controls, increased R2 durations of the blink reflex, identical to the increased R2 that occurred in our experimental Puff + Blink condition [13]. The authors speculated that this increased R2 length was the result of brain stem dysregulations of TS patients. Our findings do not rule out this possibility but they suggest a different explanation for the findings of the Smith and Lees study: the increased R2 length in patients may have resulted from extensive preceding tic behaviour disabling a normally occurring EMG habituation response. As indicated by our experimental study, it was not merely the eye blinking itself that caused these effects, but the *interaction* between the externally induced sensation and the blinking, since deviating EMG results were *not* found in the relevant control condition, where participants were instructed to blink in response to a neutral sound.

Most importantly, the study provides experimental evidence to support the speculation, first formulated more than a century ago, that tics in TS are self-perpetuating. After deliberate blinking in response to unpleasant sensations, the response to future sensations was altered, in comparison to the control group who had not been instructed to blink deliberately in response to these sensations. Thus, where in the other conditions habituation occurred to the puff condition, habituation was blocked in the crucial Puff + Blink condition. Moreover, as in the control conditions the length of the R2 response remained unaltered, in the Puff + Blink condition it was increased. A parsimonious behavioural explanation of the findings is that in the Puff + Blink condition, tic behaviour was operantly reinforced by the reduced subjective annoyance of the puff, fostering the perseverance of blinking. These findings converge with TS patients' reports that their tics decrease unpleasant sensations [5]. As discussed earlier, this is reminiscent of the mechanisms involved in the repetitive behaviour of OCD. In OCD, subjective distress gives rise to repetitive behaviour that may, temporarily and in the short term, reduce distress, which reinforces the repetition, up to the point where repetition becomes habitual and hard to resist [14]. In both OCD and TS, relief of distress by carrying out compulsions or tics may (partly) prevent habituation and maintain the disorder.

An effective treatment for both OCD and tics is exposure and response prevention (ERP), in which patients are encouraged to expose themselves to (in the case of OCD) anxiety-provoking situations [14] or (in the case of TS) premonitory urges [15,16], while at the same time resisting the urge to carry out compulsions (in OCD) or tics (in TS). The present study suggests why this treatment is effective in treating TS: ERP may stop the interference of tics with natural habituation – an interference taking place when tics are executed. This is in line with a key assumption underlying ERP, namely that if TS patients are habituated to premonitory urges, both the urge and the tic will eventually diminish [17].

The experimental findings of this study are mostly in line with results from clinical studies that examined the relationship between tic suppression and premonitory urges. Himle et al. found that tic suppression induced an immediate increase of the premonitory urge to tic, confirming the negative reinforcement view of tic function [18]. Further, recent research showed that TS patients with a greater ability of tic suppression (sustained over two hours) report less severe premonitory sensations, and vice versa [17]. Possibly, tic behaviour is subjectively rewarding on the short term, but eventually inhibits habituation.

The findings may have consequences for treatment. First, behaviour therapy focuses on the prevention of tic responses, but should also fruitfully deal with the temporal annoyance due to tic inhibition. Second, development and testing of pharmacotherapeutic interventions for tics may target premonitory urges, apart from the motor component of TS.

Limitations of this study are that this experiment has not been performed using an EEG or neuroimaging paradigm, and therefore no direct inferences can be made on the neurobiological basis of the suppression of the habituation response as found in our paradigm. While the exact neurobiological basis of TS is not yet fully understood, dysfunctional cortical-striatal-thalamic-cortical (CSTC) circuits play a crucial role [19]. Although somewhat controversial, networks of striatal neurons are supposed to become abnormally active in inappropriate contexts, leading to disinhibition of thalamocortical projections, which eventually lead to tic behaviour [19]. Thus, disinhibition in direct striatal output systems, leads to dysfunction in coherent oscillations of neuronal networks in thalamo-cortical circuits [19,20]. These networks seem to modulate sensorimotor gating as well as focused motor actions. When these networks are dysrhythmic, there may be a loss of control of sensory information and of motor action. Although the direct interplay between motor actions and premonitory urges in TS has hardly been studied, the view is commonly held that both pharmacotherapy and behavioral therapy adaptively modulate the misguided striatal and thalamocortical oscillations that are characteristic of TS, and thus influence both motor disinhibition *and* reduce the premonitory urges. This study has intended to make a start to study the interplay between premonitory urges and repeated tic behavior. Future studies may use this experimental model to study the neurobiological basis of the dysfunctional interplay between premonitory urges and tics, with the non habituated EMG responses as found in the Puff x Blink condition of this study as a starting point.

A further possible limitation is that the external stimulus used in this paradigm (the air puffs) may not be fully equivalent to the internal premonitory urges in TS. Possibly, there are subtle differences in the ways in which internal premonitory urges on the one hand and an external stimulus like an air puff on the other hand interact with tic behaviour. Once the physiological basis of premonitory urges in TS is better understood, the isomorphic qualities of this paradigm may be improved. Subsequent studies might also study the effects of *prolonged* tic behaviour (i.e., daily tic behaviour over a time span of days or weeks) to investigate whether similar mechanisms as found in this study are operant in the long term. In the present study, where participants blinked for only a very short time, no effects on perceived puff intensity were found, but EMG measures indicate an increasing sensitivity to the air puffs or, at least, a lack of habituation to the puffs. It might therefore be expected that, in the long term, eye blinking in response to airpuffs results in higher perceived intensity (and arguably also in increased annoyance) of the airpuffs.

In sum, this study supported the negative reinforcement theory of tic behaviour in TS patients. Additionally, EMG measures suggested that tic behaviour also blocked natural habituation of the blink response to air puffs (EMG amplitude), or even strengthened it (as found in the R2 component of the EMG response). Thus, these findings suggest that the motor tics in TS represent more than signs and symptoms only; while they subjectively reduce annoyance, tics themselves may ironically stimulate future tic behaviour and perpetuate or aggravate TS.

## Acknowledgments

The authors would like to thank Arne Leer for feedback and comments, and Jasper H. B. de Groot for data collection.
